# Gender Differentials in Self-Rated Health and Self-Reported Disability among Adults in India

**DOI:** 10.1371/journal.pone.0141953

**Published:** 2015-11-04

**Authors:** Jayanta Kumar Bora, Nandita Saikia

**Affiliations:** 1 Public Health Foundation of India, Gurgaon, India; 2 Centre for Study of Regional Development, Jawaharlal Nehru University, New Delhi, India; University of South Carolina, UNITED STATES

## Abstract

**Background:**

The extant literature on gender differentials in health in developed countries suggests that women outlive men at all ages, but women report poorer health than men. It is well established that Indian women live longer than men, but few studies have been conducted to understand the gender dimension in self-rated health and self-reported disability. The present study investigates gender differentials in self-rated health (SRH) and self-reported disability (SRD) among adults in India, using a nationally representative data.

**Methods:**

Using data on 10,736 respondents aged 18 and older in the 2007 WHO Study on Global Ageing and Adult Health in India, prevalence estimates of SRH are calculated separately for men and women by socio-economic and demographic characteristics. The association of SRH with gender is tested using a multinomial logistic regression method. SRD is assessed using 20 activities of daily living (ADL). Further, gender differences in total life expectancy (TLE), disability life expectancy (DLE) and the proportion of life spent with a disability at various adult ages are measured.

**Results:**

The relative risk of reporting poor health by women was significantly higher than men (relative risk ratio: 1.660; 95% confidence Interval (CI): 1.430–1.927) after adjusting for socio-economic and demographic characteristics. Women reported higher prevalence of severe and extreme disability than men in 14 measures out of a total20 ADL measures. Women aged less than 60 years reported two times more than men in SRD ≥ 5 ADLs. Finally, both DLE and proportion of life spent with a disability were substantially higher for women irrespective of their ages.

**Conclusion:**

Indian women live longer but report poorer health than men. A substantial gender differential is found in self-reported disability. This makes for an urgent call to health researchers and policy makers for gender-sensitive programs.

## Introduction

Recently, a growing body of research has looked at the gender gap in health and mortality, generally termed “the male-female health-survival paradox”[1–3]. A large body of literature confirms that women outlive men worldwide [4–6]. However, in Northern, Latin American and Caribbean countries [7–10], and in a few South Asian countries [11–12], women are more likely than men to report self-rated worse health and higher prevalence and incidence of disability and chronic morbidity.

Does this gender gap hold for India, where gender plays a crucial role from birth to death, and from nutrition to health care service utilizations? The answer is of enormous policy relevance. The literature addressing the gender gap in mortality in India is ample [13–16]. Unlike in the developed world, Indian females do not enjoy survival advantages over males in every age group. More recent studies find that the female infant mortality rate in many states of India is higher than the male infant mortality rate [17]. India still has a high volume of excess female deaths at infant and child age (1–4 years) due to discriminatory care at home, discriminatory health-care seeking and selective termination of female fetuses [18]. Despite this, Indian females enjoy a higher life expectancy at birth than males and the male-female gap in survivorship favours adult and elderly females.

Despite this important role of gender in health and mortality, few studies address the gender difference in self-rated health (SRH) [12,19–21] and in self-reported disability (SRD) in India [22–24]; these debate the gender difference in SRH and SRD side by side and arrive at different conclusions. While some studies [21,25] demonstrate that the gender differential in SRH persists even after adjusting socio-economic variables, other studies show that the gender differential in self-assessed health turns in favour of women when adjusted for the role of socio-economic factors [19]. It is found that women tend to report worse health than men, especially among the socio-economically advantaged group [25], and socio-economic status contributes significantly to this gap [19]. Studies on gender difference in SRD also reveal that, in general, women report higher disability condition than men [22–23], but the gender difference in disability may not be significant in the context of South India [24].

Self-rated health (SRH) is a widely used measure based on a person's self-assessment of his/her status in response to the question “In general, how would you rate your health today?” [26–28]. Although SRH overlooks the concern of interpersonal incomparability; it is a remarkably reliable measure that is consistent with the actual health status of the respondents [26,29]. Self-rated health has been used to assess the health status of populations and predict health outcome, survival, impending morbidity and death [26,29–31]. Self-reported disability, another SRH measure, is extensively used to assess disability and is considered a reflection of true disability among the elderly population [32–33]. It is defined as functional limitations in activities of daily living involving bathing, eating, a transfer from bed to chair, use of the toilet, etc. [33]. Numerous earlier studies demonstrated empirically that data on SRH and SRD is consistent with both performed measures and medical diagnosis [32,34–35]. Earlier studies confirmed that both men and women report their disability accurately, and the higher prevalence of reported functional problems among women is perhaps a reflection of true disability for most disability measures [32]. A five-year follow-up survey of the Study on Global Ageing and Adult Health (SAGE) conducted by the World Health Organization (WHO) multi-country study on global ageing and adult health in rural India found that bad or very bad self-rated health was a strong predictor of mortality for persons aged above 50, even after controlling for socio-demographic and disability measures [26].

The purpose of the present study is to examine the gender differential in SRH and SRD among older adults in India. Our study extends the extant knowledge in several directions. First, we combine the analysis of gender difference in SRH and SRD to present a coherent picture of the gender difference in health status among adults in India. Second, we investigate gender difference in total life expectancy (TLE), disabled life expectancy (DLE) and disabled free life expectancy (DFLE) to demonstrate the health disadvantage of women despite survival advantage in adult age. Finally, unlike most of the previous studies on SRD in India, our study is based on a nationally representative survey, indicating a generalization of results at national level. The findings on gender inequality in adult health might prove useful for health planners and policy makers, especially in countries subject to rapid health transition.

## Data and Methods

### 2.1. Ethics statement

We used cross-sectional WHO SAGE survey data, approved by the Ethics Review Committee of the WHO, Geneva and the International Institute for Population Sciences, Mumbai (IIPS) implementing the national SAGE survey. Before conducting the interviews, respondents participating in this survey were given a “Respondent information Form”, which detailed the giving details of the survey purpose, methods and data collection procedure involved in this survey. Finally, written informed consent was taken from the respondents of aged 21 and older. For participants under 21 years old of age, a parent or guardian also signed in the consent form.

### 2.2. Data Description

We used data primarily from the SAGE, carried out by the IIPS in 2007–08 under the WHO multi-country study on global ageing and adult health. The main objective of this study was to obtain reliable, valid and comparable data on levels of health across a range of key domains for adult populations aged 50-plus in nationally represented samples. The study aimed also to supplement and cross-validate self-reported measures of health by anchoring a vignette approach to improving the comparability of self-reported measures for selected health domains [36–37].

The first wave of the SAGE was implemented in six states selected to ensure a nationally representative sample—Assam, Karnataka, Maharashtra, Rajasthan, Uttar Pradesh and West Bengal. The same primary sampling units (PSUs) and households covered in the 2003 World Health Survey (WHS) are comprised the baseline sample for SAGE Wave 1 India in 2007–08. The SAGE Wave 1 India included a total sample of 12,198. Out of 12,198 interviews, 10,736 interviews were completed, 494 interviews were partially completed and the rest were either refused or missed. Our analysis is based on 10,736 completed interviews, of which about 58.2 percent belong to the 50+ age group.

Through face-to-face interviews, information was collected on the physical characteristics of the dwelling or household; a household roster, including the sex, age, marital status, education, and care needs of each household member; cash and non-cash transfers into and out of the household; household income and expenditure; work history and benefits; health and health behaviours; chronic conditions; health care utilization; social networks; subjective well-being and quality of life; and on the impact of caregiving. The health status of individuals was also assessed with the help of the following biomarkers: anthropometry (weight, height, body mass index, waist-to-hip ratio); physical tests (timed walk, hand grip strength, lung function, vision tests, blood pressure); cognition tests (verbal fluency, immediate and delayed verbal recall, digit span); and blood tests (from consenting respondents, to test for anaemia, diabetes, and cardiovascular disease). A detailed description of this data can be found in the India National Report of the SAGE [36].

### 2.3. Measures

Self-rated health was defined by the answer to the question “In general, how would you rate your health today?” The options to answer this question were available on a five-point Likert scale: very good, good, moderate, bad and very bad. In our analysis, we converted the responses from a five-point scale to a three-point scale by combining very good and good to “good”, moderate to “moderate” and very bad and bad to “poor”. Self-reported disability in 20 activities of daily living (ADL, defined as having severe or extreme difficulties performing activities of daily living) measures—addressing mobility, self-care, pain and discomfort, cognition, interpersonal activities, sleep and energy, affect and vision were used to measure disability. For example, the first question related to mobility is “Overall in last 30 days, how much difficulty did you have with moving around?” The options to answer this question were available on a five-point Likert scale: none, mild, moderate, severe and extreme or cannot do. If the answer from the respondent “severe” or “extreme or cannot do” in moving around, it is defined as a self-reported disability in moving around ADL. The details of the questions used to define self-reported disability are given in S1 Appendix.

We analyzed gender differentials in self-rated health by a number of socio-economic and demographic variables, including age, gender, education (primary school or less, secondary school completed, tertiary or higher education); wealth quintiles of household, (Q1 lowest to Q5 highest), religion (Hindu, Muslim and Others); ethnicity (Others, Scheduled Caste (SC) and Scheduled Tribe (ST)); and marital status (currently married, never married and separated/divorced/widowed).

### 2.4. Statistical Methods

To estimate the prevalence of SRH and SRD by gender, we used STATA S.E. 10.0 (STATA Corp., Inc., College Station, TX). We performed chi-square test to assess whether there were significant gender differences in the sample by socio-economic and demographic factors and also in self-reported disabilities. To estimate DFLE and DLE, the Sullivan Method [38] was applied. We used the proportion of disability by age and sex on Sample Registration System (SRS)[39] life tables of India for the period 2006–2010. The SRS, a dual vital registration system in India covering six million of population in India, provides the most reliable estimates of vital rates at national and sub-national levels (state categorized by rural-urban) since the 1970s. A detailed description of SRS data can be found elsewhere [40]. We used multinomial logistic regression to assess the adjusted effect of gender on SRH. The outcome variable has three categories: self-reported good, moderate and poor health. We estimated the relative risk of (1) poor health versus good health and (2) moderate health versus good health. Age was estimated as a continuous independent variable. The gender, education and wealth quintiles of a household, religion, ethnicity and marital status were considered as categorical independent variables. A p-value less than or equal to 0.05 was considered significant. The evidence for multicollinearity was assessed by the multicollinearity diagnostic (variance inflation factor or VIF). All VIFs were less than 2, indicating that the assumption of reasonable independence among predictor variables was met. A total of 113 missing cases (1 case in age, 71 cases in wealth index and 41 cases in ethnicity) were excluded from the multinomial logistic regression analysis.

## Results

### 3.1. Self-rated Health (SRH)

Table 1 presents gender differentials in self-rated health by socio-economic and demographic characteristics. The figures presented in the table are the percentage of men and women reporting self-rated health, categorized into good, moderate and poor health. In general, 15.1% of Indian women rated their health as “poor” compared to15.2% of Indian men. Most findings in this table are in the expected direction. For example, as age increases, the percentage of people reporting poor health increases (men: 3.8% to 38.4%; women: 4.7% to 44.6%); urbanites enjoy better health than rural people (45.4% urban men report good health against37.7% rural men; 40.7% urban women report good health against 38.0% rural women); as education increases, the percent of people reporting good health increases (Men: 28.4% to 56.9%; Women: 30.8% to 61.3%), as household wealth index increases from poorest to least poorest quintile, self-rated health improves (Men:32.6% to 47.8%; Women:34.5% to 45.0%). Finally, people belonging to deprived social groups like Muslims, STs and SCs reported poor self-rated health. Table 1 also presents the *p-*value of chi-square test examining gender difference in socio-economic and demographic variables. Except for a few predictors (wealth index, religion and ethnicity), *p*-values were always significant, indicating a clear existence of gender difference in the predictor variables.

**Table 1:** Percent of men and women reporting self-rated health by socio-economic and demographic characteristics, 2007–2008, India.

	Men				Women				
	N	Good	Moderate	Poor	N	Good	Moderate	Poor	*p-*value^1^
**Age**									0.000
18–29	262	70.2	26.0	3.8	1,278	68.2	27	4.7	
30–39	345	62.3	31.0	6.7	1,259	47.3	42.3	10.4	
40–49	394	52.0	39.1	8.9	951	37.8	50.7	11.6	
50–59	1,352	43.0	46.0	11.0	1,486	27.2	55.9	17.0	
60–69	1,099	28.2	53.5	18.3	1,021	22.7	54.8	22.5	
70–79	570	22.9	48.9	28.1	429	14.9	50.2	33.3	
80+	152	15.2	46.4	38.4	138	8.6	46.8	44.6	
**Place of residence**									0.000
Urban	974	45.4	43.7	10.9	1,732	40.7	48	11.4	
Rural	3,199	37.7	45.7	16.6	4,831	38	45.6	16.4	
**Education**									0.000
No education	1,208	28.4	51.1	20.5	3,577	30.8	50.4	18.9	
Primary	1,266	34.4	48.3	17.3	1,508	38.7	47	14.3	
Secondary	671	42.6	44.9	12.5	678	53.8	37.8	8.4	
High school & above	1,028	56.9	34.8	8.3	800	61.3	33.6	5.1	
**Wealth Index**									0.451
Q1 poorest quintile	694	32.6	48.3	19.2	1,176	34.5	44.9	20.6	
Q2	804	34.2	47.8	18	1,236	37.7	44.6	17.7	
Q3	786	40.3	40.7	19	1,253	37	47.6	15.4	
Q4	902	40.2	47.8	12	1,367	38.3	49.6	12.1	
Q5 least poor quintile	961	47.8	42.6	9.7	1,486	45	44.5	10.6	
**Religion**									0.600
Hindu	3,498	40.3	45.1	14.7	5,549	39.9	46.2	14.0	
Muslim	518	33.8	44.8	21.4	776	31.6	45.9	22.6	
Other	157	42.0	50.3	7.6	238	34.9	49.2	16.0	
**Ethnicity**									0.977
Scheduled Tribe (ST)	286	37.4	46.2	16.4	454	39.2	41.9	18.9	
Scheduled Caste (SC)	728	38.9	44.0	17.2	1,136	38.2	45.0	16.7	
Other	3,142	39.9	45.4	14.7	4,949	38.8	47.0	14.3	
**Marital Status**									0.000
Currently married	3,620	39.6	45.7	14.7	4,753	40.5	46.8	12.7	
Never married	183	60.7	31.7	7.7	415	74.2	23.1	2.7	
Separated/divorced/widowed	370	27.8	47.8	24.3	1,395	22.1	51.1	26.8	
**Total**	**4173**	**39.5**	**45.2**	**15.2**	**6563**	**38.7**	**46.2**	**15.1**	

^1^ indicates the *p* value of chi-square test assessing association between gender and socio-economic and demographic characteristics.

The most noticeable finding in Table 1 is the presence of clear gender differences in self-rated health by socio-economic and demographic characteristics. Women from each age group report systematically poorer self-rated health than men. Interestingly, as age increases, the gender difference in self-rated health also increases. While a higher percentage of urban men than urban women rate their health as “good”, there is no difference in self-rated health between rural men and women. Likewise, except those in the deprived categories (say, belonging to rural, low educated, household with poor wealth quintile and scheduled tribes) all women rated their health worse than men.

### 3.2. Self-rated disabilities in activities of daily living (ADL)

Table 2 reports the prevalence estimates of SRD in 20 ADL measures by gender in India for 2007–2008. The most striking finding is that out of twenty ADL measures addressing mobility, self-care, pain/discomfort, cognition, interpersonal activities, sleep/ energy, affect and vision, women reported systematically higher prevalence of severe and extreme disability than men in fourteen measures. Among all these measures, the gender difference in self-rated disabilities is more pronounced in three components: pain and discomfort, interpersonal relationship and affects. The *p-*values of chi-square test examining gender difference in self-reported disabilities again indicate a clear existence of gender difference in performance of activities of daily living.

**Table 2:** Prevalence Estimates (in percentage) of self-reported disabilities (severe and extreme difficulty combined together) by gender.

	Male		Female		
Variables	N	%	N	%	*p*value^2^
**Mobility**					
Moving around	400	9.6	655	10	0.503
Vigorous activities	1,202	28.8	1,764	26.9	0.030
**Self-care**					
Self care	80	1.9	157	2.4	0.102
Taking care of and maintaining general appearance	73	1.8	127	1.9	0.488
Staying by yourself	342	8.2	446	6.8	0.007
**Pain and Discomfort**					
Bodily aches or pains	470	11.3	1,028	15.7	0.000
Bodily discomfort	480	11.5	927	14.1	0.000
Difficulty because of pain	442	10.6	832	12.7	0.001
**Cognition**					
Concentrating or remembering things	306	7.3	618	9.4	0.000
Learning a new task	543	13	1,008	15.4	0.001
**Interpersonal Relationship**					
Personal relationships	172	4.1	425	6.5	0.000
Dealing with conflicts and tensions	257	6.2	541	8.2	0.000
Making new friendships or maintaining current friendships	188	4.5	372	5.7	0.008
Dealing with strangers	431	10.3	685	10.4	0.857
**Sleep and Energy**					
Having difficulties with sleeping	356	8.5	679	10.4	0.002
Not feeling rested and refreshed	403	9.7	730	11.1	0.016
**Affect**					
Feeling sad, low or depressed	245	5.9	548	8.4	0.000
Worry or anxiety	444	10.6	939	14.3	0.000
**Vision**					
Difficulties in seeing and recognizing an object across the road	432	10.4	778	11.9	0.016
Difficulties in seeing and recognizing an object at arm's length	412	9.9	622	9.5	0.498

^2^ indicates the *p* value of chi square test assessing association between gender and self-reported disabilities in activities of daily living.

**Fig 1:**
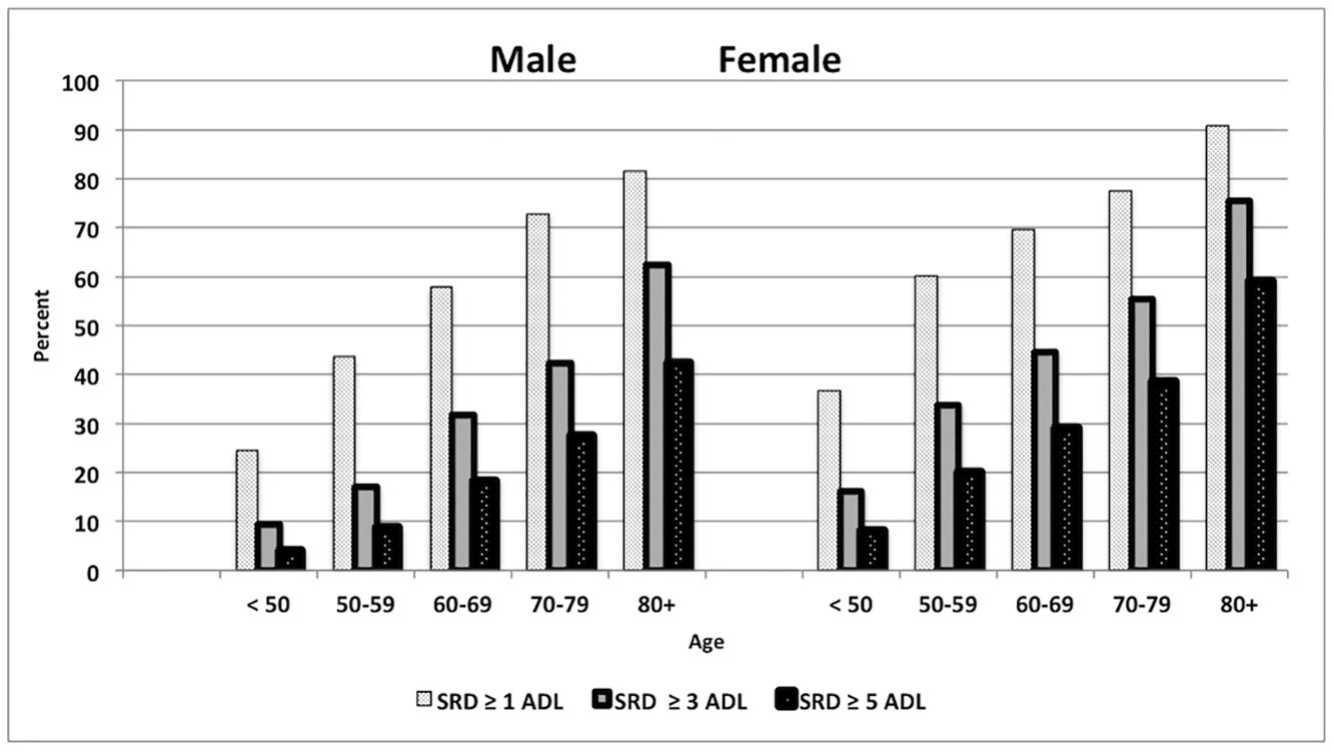
Prevalence Estimates (in percentage) of self-reported disabilities by gender and age groups, India, 2007–2008.

Table 3 and Fig 1 further present prevalence estimates (in percentage) in at least one, three and five self-reported disabilities by gender and age groups in India for 2007–2008. Table 3 and Fig 1 clearly suggest that a higher proportion of women suffer from disabilities in ADL in all adult age groups. However, the gender differential was the highest in the age group less than 50 and 50–59, with the percent of women reporting more than five disabilities being two times more than their male counterparts (Male: 3.9% against 8.1% female in age group <50; Male: 8.7% against 20.0% female in age group 50–59) etc.

**Table 3:** Prevalence Estimates (in percentage) of self-reported disabilities by gender and age groups, India, 2007–2008.

Gender and Age (yrs) Groups	Sample Size	SRD ≥ 1 ADL	SRD ≥ 3 ADL	SRD ≥ 5 ADL
**Males**				
18–50	1001	24.6	9.4	3.9
50–59	1352	43.6	17.1	8.7
60–69	1099	57.8	31.7	18.2
70–79	570	72.8	42.3	27.4
80+	151	81.5	62.3	42.4
**Females**				
18–50	3488	36.6	16.1	8.1
50–59	1486	60.2	33.7	20.0
60–69	1021	69.7	44.5	29.1
70–79	429	77.4	55.5	38.5
80+	139	90.7	75.5	59.0
**Total**				
18–50	4489	33.9	14.6	7.2
50–59	2838	52.3	25.8	14.6
60–69	2120	63.5	37.8	23.4
70–79	999	74.8	48.0	32.1
80+	290	85.9	68.6	50.3

SRD = Self-reported Disability, ADL = Activities daily living

Table 4 demonstrates total life expectancy (TLE), DFLE, DLE and percent of life with disability (DLE/TLE) by age and gender, calculated for at least one, three and five disabilities out of twenty disabilities as shown in Table 2. After age20, women are expected to live longer than men at each age. On average, women live two years longer than men, and the gender gap (women versus men) reduces from 4.0 years at age 20 to 0.6 years at age 80. However, women are also expected to live more years with disability, as DLE for women for any number of disabilities is higher than DLE for men at each age. Interestingly, the gender disparity in DLE is far more pronounced than the gender disparity in total life expectancy and varies greatly by age. For example, the gender difference in DLE calculated for at least one self-disability is a high as 9.8 years at age20; it drops to 5.2 years at age 50 and to 3.2 years at age 60. The DLE calculated for at least one self-rated disability shows that both Indian men and women spend a considerable proportion of their life with at least one disability; for example, at age 50, men and women are expected to live 57.3% and 70.5% of their life with at least one type of disability respectively. Once again, women spend a higher proportion of their life with poorer health status.

**Table 4:** TLE, DFLE and DLE (in years) by Gender and Age, India, 2007–2008.

	Age 20		Age 50		Age 60		Age 70		Age 80	
	Male	Female	Male	Female	Male	Female	Male	Female	Male	Female
TLE	49.9	53.9	23.9	26.8	16.5	18.6	10.7	12	6.9	7.5
Limitation in at least one SRD										
DLE	18.8	28.6	13.7	18.9	10.9	14.1	8.2	9.9	5.7	6.8
DFLE	31.1	25.3	10.2	7.9	5.6	4.4	2.6	2.1	1.2	0.7
DLE/TLE (%)	37.8	53.1	57.3	70.5	66.3	76.2	76.1	82.4	83.2	90.9
Limitation in at least three SRD										
DLE	9.1	16.5	7.4	12.5	6.6	10.0	5.3	7.5	4.5	5.7
DFLE	40.8	37.3	16.5	14.3	9.9	8.6	5.4	4.5	2.4	1.8
DLE/TLE (%)	18.2	30.7	31.0	46.5	40.1	53.7	49.7	62.4	65.5	75.4
Limitation in at least five SRD										
DLE	4.9	10.3	4.5	8.4	4.1	7.0	3.5	5.5	3.1	4.4
DFLE	45.0	43.5	19.5	18.4	12.4	11.6	7.2	6.5	3.8	3.1
DLE/TLE (%)	9.8	19.2	18.6	31.4	25.1	37.7	33.0	45.8	45.0	58.8

TLE-total life expectancy, DLE-disabled life expectancy, DFLE-disabled free life expectancy, SRD: Self-rated disability

The magnitude of DLE and percentage of life with disability reduced to almost half and one-fourth when we calculated the same for at least three and five self-rated disability respectively. Nevertheless, we observed similar kind of gender disparity in TLE, DFLE, DLE and percent of life with a disability.

Table 5 presents the results of the multinomial logistic regression. The majority of the associations found in Table 5 are consistent with the findings of Table 2. The relative risk ratio (RRR) of having poor health versus good health increases as age increase (RRR: 1.90; CI: 1.81–2.00); decreases as wealth of the household increases; is higher among Muslims (RRR: 2.04; CI: 1.69–2.47); is lower among the never married (RRR:0.64; CI:0. 41–0.99);and is higher among the separated or divorced (RRR:1.30; CI: 1.09–1.55). All these relationships are statistically significant. Similar results are found in the RRRs of having moderate health versus good health, as shown in Table 5. The most noticeable point in Table 5 is that females have significantly higher relative risk than men of having poor health versus good health (RRR: 1.69; CI: 1.46–1.97), and of having moderate health versus good health (RRR: 1.39; CI: 1.25–1.55). Thus, even after controlling for relevant socio-economic variables, women are more likely than men to report poor or moderate health.

**Table 5:** Multinomial Logistic regression: Predictors of self-rated health among adults in India, 2007–2008.

	Poor Health (Adjusted)			Moderate Health (Adjusted)		
Variables	RRR	[95 CI]	P>|z|	RRR	[95 CI]	P>|z|
**Age (in years)**	1.905	(1.810–2.006)	0.000	1.453	(1.403–1.506)	0.000
**Gender**						
Male^®^						
Female	1.697	(1.461–1.973)	0.000	1.397	(1.255–1.555)	0.000
**Place of residence**						
Rural^®^						
Urban	0.794	(0.675–0.934)	0.005	1.051	(0.945–1.169)	0.360
**Education**						
No education^®^						
Primary	1.085	(0.926–1.271)	0.315	0.929	(0.829–1.041)	0.205
Secondary	0.897	(0.711–1.133)	0.362	0.753	(0.649–0.873)	0.000
High school & above	0.574	(0.449–0.734)	0.000	0.538	(0.462–0.627)	0.000
**Wealth Index**						
Q1 poorest quintile^®^						
Q2	0.826	(0.680–1.004)	0.055	0.914	(0.789–1.058)	0.229
Q3	0.814	(0.667–0.992)	0.042	0.904	(0.778–1.050)	0.186
Q4	0.610	(0.494–0.753)	0.000	0.997	(0.858–1.159)	0.971
Q5 least poor quintile	0.442	(0.353–0.553)	0.000	0.771	(0.657–0.903)	0.001
**Religion**						
Hindu^®^						
Muslim	2.045	(1.692–2.473)	0.000	1.212	(1.046–1.405)	0.011
Other	0.906	(0.635–1.291)	0.584	1.173	(0.929–1.480)	0.180
**Ethnicity**						
Other^®^						
ST	1.395	(1.093–1.780)	0.007	0.984	(0.821–1.179)	0.863
SC	1.220	(1.029–1.448)	0.022	0.960	(0.849–1.086)	0.524
**Marital Status**						
Currently married^®^						
Never married	0.644	(0.415–0.998)	0.049	0.744	(0.604–0.916)	0.005
Separated/divorced/widowed	1.308	(1.099–1.556)	0.002	1.009	(0.875–1.162)	0.906

Good Health is the base outcome; RRR = Relative Risk Ratio, Ref category ^**®**^

## Discussion and Conclusion

Our first objective was to contribute to the debate over the gender difference in SRH in India. We found that, in general, Indian adult women report poorer health than Indian adult men. This finding is consistent with the findings of studies conducted in other countries [41– 43] and in India [12,19,21,25, 44–46]. This study found that the gender difference in SRH persisted even after adjusting the effect of the socio-economic condition. This is contrary to the findings of a previous study [19]. The finding of this study, however, is consistent with the previous findings [20–21] that health disadvantages among women could not be explained by the difference in demographics and socio-economic characteristics.

There are a few explanations why women live longer than men but have poorer health. Several studies exhibit that this is due to biological, social and behavioral factors [47–50]. Researchers have suggested that two factors in particular contribute the excess mortality of men. First, women are more likely than men to adopt preventative health behaviors, such as routine annual visits to a physician for a check-up. Men are more likely to engage in risky behaviours, such as excessive drinking, drunken driving, illegal drug use, physical fights and violence and high tobacco consumption [48,51–55]. Early in life, boys are more susceptible to death than girls due to biological reasons [56–57]. In adolescence, youth and adulthood, men die from exposure to risks linked to their social and behavioural characteristics [48]. Studies argued that poorer health among women is due to biological as well as behavioural factors. Some studies discuss that while women suffer more than men, female ailments tend to be less lethal biologically [7,58]. Some other studies refer to the over-reporting of worse health among women [59–60]. In addition, women’s longer life expectancy also influences male-female differences in health status [3]. It is showed that larger the female excess in longevity, larger the female excess in the proportion of life in poor health [3].

Our second objective was to extend the analysis to SRD and its connotation with life expectancy at various stages of adulthood. All three measures of SRD (prevalence estimates of SRD in more than one, three and five ADL measures, corresponding DLEs and proportion of life spent with disability at various stages of life) revealed that Indian women are subject to higher levels of disability irrespective of their age. Notably, the gender gap in disability is found to be the highest in difficulty in more than five ADLs in the age groups before age 60 (women reported two times more than men). Our findings are consistent with the common wisdom that the gender difference in disability is substantial, especially in ADL measures [61– 64]. Future research should focus more on the unmet need of personal care among disabled adults whether gender plays an important role in caregiving to differential exists in proving care and treatment.

In the past several decades, life expectancy has increased in India; there are now over 192 million people aged 50 or older [39]. As a consequence of the rapid ageing of the population, the central theme of health research in India has expanded beyond longevity towards understanding the burden of disease and disability, particularly among adults. Longevity is greater among females than males, especially at adult ages, in India—as in all other countries. Does the survival advantage of females translate to their poorer health? Answering this question is of enormous importance for future health care needs and policy. A few studies address the gender differential in health and disability among older adults in India [20–21,24–25,65–68], but this study extends the knowledge by examining SRH and SRD together. By using nationally representative survey data, this study first investigated the role of gender in self-rated health among adults in India after controlling for socio-economic and demographic characteristics, and examined gender differential in SRD in performing twenty ADLs (severe and extremely difficulty) and thereafter revealed the linkage between survival and SRD measured by TLE, DFLE, DLE and percent of life spent with self-reported disability.

This study suggests that in India, as in other parts of the world, women bear the greater burden of disability in their adult age. In the absence of governmental support, the problem might be acute, especially among marginalized women living alone, widowed and poorly off socio-economically. The findings of this study strongly suggest the need for a coherent and gender sensitive health agenda for ageing populations in India. We also suggest future in-depth studies focused on the complex gender dynamics in health and disability in India at a regional level.

## Limitations

The present study has a few limitations. First, we could not throw light on the regional dimensions of gender differentials in SRH and SRD because the sample size at the regional level is inadequate. Previous studies have documented the regional divide in gender differentials in health outcomes in India [44,69]. Neither could we test the relationship between female excess longevity and female excess in the proportion of life in poor health using time trend data. Second, we could not analyze the trend of gender differentials in DLE in India due to lack of such data. In the absence of it, we again could not test whether increasing SRD by women is a function of increasing survivorship or that women naturally report poorer health even in the absence of acute gender differential in survivorship.

## Supporting Information

S1 Appendix List of the questions used to define self-reported disability. (DOCX)
