# Supplementary material for: Gender Differentials in Self-Rated Health and Self-Reported Disability among Adults in India
Source: PLoS One. 2015 Nov 4;10(11):e0141953. doi: 10.1371/journal.pone.0141953 (PMC4633186; doi:10.1371/journal.pone.0141953)
Supplement: S1 Appendix — (DOCX) [file pone.0141953.s001.docx]

**S1 Appendix : List of the questions used to define self-reported disability**

Now I would like to review the different functions of your body. When answering these questions, I would like you to think about the last 30days, taking both good and bad days into account. When I ask about difficulty, I would like you to consider how much difficulty you have had, on average, in the last 30days, while doing the activity in the way that you usually do it. Let me remind you, by difficulty I mean requiring increased effort, discomfort or pain, slowness or changes in the way you do the activity.

INTERVIEWER: Read and show scale to respondent.

MOBILITY

| Overall in the last 30days, how much difficulty did you have… | | NONE | MILD | MODERATE | SEVERE | EXTREE/CANNOT  DO |
| --- | --- | --- | --- | --- | --- | --- |
| Q2002 | …with moving around? | 1 | 2 | 3 | 4 | 5 |
| Q2003 | …in vigorous activities ('vigorous activities' require hard physical effort and cause large increases in breathing or heart rate)? | 1 | 2 | 3 | 4 | 5 |

INTERVIEWER: Use Show card if needed for mobility.

SELF-CARE

| Over all in the last 30days, how much difficulty did you have… | | NONE | MILD | MODERATE | SEVERE | EXTREME/CANNOT DO |
| --- | --- | --- | --- | --- | --- | --- |
| Q2004 | …with self-care, such as bathing/washing or dressing yourself? | 1 | 2 | 3 | 4 | 5 |
| Q2005 | …in taking care of and maintaining your general appearance (for example, grooming, looking neat and tidy)? | 1 | 2 | 3 | 4 | 5 |
| Q2006 | …in staying by yourself for a few days (3 to 7days)? | 1 | 2 | 3 | 4 | 5 |

PAIN AND DISCOMFORT

| Over all in the last 30 days,… | EXTREME/ NONE MILD MODERATE SEVERE CAN NOT  DO | |
| --- | --- | --- |
| Q2007…how much of bodily aches or pains did you 1 2 3 4 5  have | | |
| Q2008 …how much bodily discomfort did you have?  1 2 3 4 5 | | |
| IfQ2007andQ2008areboth=1,"None"…………………………………………………………..…. | | Q2010 |
| Q2009 …how much difficulty did you have in your  daily life because of your pain? 1 2 3 4 5 | | |

COGNITION

| Overall in the last 30days, how much difficulty… | | NONE | MILD | MODERATE | SEVERE | EXTREME/  CAN NOT  DO |
| --- | --- | --- | --- | --- | --- | --- |
| Q2010 | …did you have with concentrating or remembering things? | 1 | 2 | 3 | 4 | 5 |
| Q2011 | …did you have in learning a new task (for example, learning how to get to a new place, learning a new game, learning a new recipe)? | 1 | 2 | 3 | 4 | 5 |

INTERPERSONAL ACTIVITIES

| Overall in the last 30days, how much difficulty did you have,… | | NONE | MILD | MODERATE | SEVERE | EXTREME/  CAN NOT DO |
| --- | --- | --- | --- | --- | --- | --- |
| Q2012 | …with personal relationships or participation in the community? | 1 | 2 | 3 | 4 | 5 |
| Q2013 | …in dealing with conflicts and tensions with others? | 1 | 2 | 3 | 4 | 5 |
| Q2014 | …with making new friendships or maintaining current friendships? | 1 | 2 | 3 | 4 | 5 |
| Q2015 | …with dealing with strangers? | 1 | 2 | 3 | 4 | 5 |

SLEEP AND ENERGY

| Overall in the last 30days, how much of a problem did you… | | NONE | MILD | MODERATE | SEVERE | EXTREME/CAN NOT DO |
| --- | --- | --- | --- | --- | --- | --- |
| Q2016 | …have with sleeping, such as falling asleep, waking up frequently during the night or waking up too early in the morning? | 1 | 2 | 3 | 4 | 5 |
| Q2017 | …have due to not feeling rested and refreshed during the day (for example, feeling tired, not having energy)? | 1 | 2 | 3 | 4 | 5 |

AFFECT

| Overall in the last 30days, how much of a problem did you have… | | NONE | MILD | MODERATE | SEVERE | EXTREME/ CAN NOT DO |
| --- | --- | --- | --- | --- | --- | --- |
| Q2018 | …with feeling sad, low or depressed? | 1 | 2 | 3 | 4 | 5 |
| Q2019 | …with worry or anxiety? | 1 | 2 | 3 | 4 | 5 |

VISION (Respondent should answer, as when wearing glasses/contact lenses if used)

| Q2023 | In the last 30days, how much difficulty did you have in seeing and recognizing an object or a person you know across the road (from a distance of about 20 meters)? | 1. NONE 2. MILD 3. MODERATE 4. SEVERE 5. EXTREME/CANNOT DO |
| --- | --- | --- |
| Q2024 | In the last 30days, how much difficulty did you have in seeing and recognizing an object at arm's length (for example, reading)? | 1. NONE 2. MILD 3. MODERATE 4. SEVERE 5. EXTREME/CANNOT DO |
